# Dosimetry, Optimization and FMEA of Total Skin Electron Irradiation (TSEI)

**DOI:** 10.1016/j.zemedi.2021.09.004

**Published:** 2021-11-02

**Authors:** Maya Shariff, Willi Stillkrieg, Michael Lotter, Daniel Lohmann, Thomas Weissmann, Rainer Fietkau, Christoph Bert

**Affiliations:** a Department of Radiation Oncology, Universitätsklinikum Erlangen, Friedrich-Alexander-Universität Erlangen-Nürnberg, Universitätsstraße 27, 91054, Erlangen, Germany; b Comprehensive Cancer Center Erlangen-EMN (CCC ER-EMN), Erlangen, Germany

**Keywords:** Total Skin Electron Irradiation (TSEI), FMEA, HDRE, Quality assurance, Dosimetry, Mycosis fungoides

## Abstract

**Purpose:**

Total Skin Electron Irradiation (TSEI) is a method for treating malignant cutaneous T-cell lymphomas. This work aims to implement and optimize the total skin technique established at Strahlenklinik Erlangen, Germany on two new linear accelerators and to quantify the risks using failure mode and effects (FMEA) analysis.

**Material and methods:**

TSEI is performed at a VersaHD accelerator (Elekta, Stockholm) with 6 MeV in the “high dose rate mode” HDRE and a nominal field size of 40 × 40 cm^2^. To reach the entire skin surface, the patients perform 6 different body positions at a distance of 330 cm behind an acrylic scatter plate, with two overlapping irradiation fields being radiated at 2 gantry angles per position. The irradiation technique was commissioned according to the recommendation of AAPM report 23. With the help of a reference profile at 270°, 2 gantry angles were calculated, which in total resulted in an optimal dose distribution. This was metrologically verified with ion-chamber measurements in the patient's longitudinal axis. The influence of the shape of the acrylic scatter plate and the distance between the acrylic scatter plate and patient was determined by measurements. The dose homogeneity was verified using an anthropomorphic disc phantom equipped with GafChromic films. The workflows and failure modes of the total skin technique were described in a process map and subsequently quantified with a FMEA analysis.

**Results:**

An optimal dose distribution is achieved at a distance of SSD = 330 cm, using the gantry angles 289° and 251°. The previously used segmented acrylic scatter plate was replaced by a flat plate (200 × 120 × 0.5 cm^3^), which is placed at a distance of 50 cm in front of the patient. The densitometric evaluation of the GafChromic films in the anthropomorphic disc phantom revealed an expected dose distribution of 3 Gy at a depth of up to 1.5 cm below the skin surface, with a homogeneity of ±10% over the phantom's longitudinal axis. By FMEA a maximum risk priority number of 30 was determined.

**Conclusion:**

Based on the calculations and measurements performed on the new accelerators as well as the risk analysis, we concluded that total skin therapy can be implemented clinically.

## 1 Introduction

Total Skin Electron Therapy (TSEI) is mainly used for the treatment of mycosis fungoides or Sezary syndrome, a rare T-cell lymphoma, with localization in the first millimetres of the skin [1]. The entire skin surface is irradiated homogeneously (target: ±10%) to a defined depth with electrons of high dose rate ensuring that the body exposure is only 1–3% of the focal dose [2]. Only with electron therapy focal doses of more than 30 Gy can be applied while keeping the whole body exposure at tolerable levels [3]. The technique was first developed at Stanford University in 1950 and has been further modified since then, as shown in AAPM Report No. 23 [4]. A modified Stanford technique according to Müller et al. [3] has been performed at the Strahlenklinik Erlangen since 1984. Till 2019, the treatment was performed at a Primus linear accelerator (Siemens, Germany).

To be able to cover the entire skin surface, patients are typically positioned at an extended focal surface distance of 3 to 4 meters. Two electron fields with a nominal field size of about 40 × 40 cm^2^ are used, which are tilted by about ±20° against the horizontal axis, so that the cranial and caudal areas are covered once [5] to reach a total dose of 30 Gy at $z_{ref}=\left(0.6R_{50}-0.1\right)/\text{cm}$ according to IAEA TRS-398 [6] which for low energy electrons is nearly identical to d_max_. Additionally, there are some studies for low-dose total skin electron beam therapy with a median dose of 12 Gy [7], which improved disease symptoms and emotional domains of patients’ quality of life.

With the replacement of two linear accelerators, the irradiation technique for total skin therapy had to be adapted to a new linear accelerator model, the adapted technique had to be commissioned, and constancy test characteristics had to be defined according to AAPM Report No. 23 [4], IAEA TRS-398 [8], and AAPM TG51 [9]. The dosimetry of this technique requires measurements and quality assurance methods that cannot be performed under reference conditions according to IAEA TRS-398 [8]. Accordingly, comparable alternatives had to be identified and validated. To quantify the risks associated with the implementation, all the necessary workflow steps and the failure modes associated with them were defined and a failure mode and effects (FMEA) analysis was subsequently carried out.

## 2 Material and methods

The Strahlenklinik Erlangen is equipped with two identical VersaHD (Elekta, Stockholm) linear accelerators (linacs), which have a special High Dose Rate Electron (HDRE) mode emitting electrons of a nominal energy of 6 MeV at a 10-fold increased dose rate compared to the standard electron mode. At a source to surface distance (SSD) of 100 cm, dose rates of 30–40 Gy/min can be generated at the maximum of the percentage depth dose (PDD) curve. For HDRE irradiation, a special open frame is required, producing a 40 × 40 cm^2^ field in the isocentre. The operation of the HDRE mode is also secured by an additional key switch.

In this work first the beam characteristics are measured both at the reference distance of 100 cm and in the extended SSD used for treatment. Furthermore, the influence of the production-related edges of the previously used segmented acrylic scatter plate on scattering is investigated. To achieve a homogeneous profile, two open fields in superposition are necessary [1]. Since the measurement of every possible angle combination would be too time-consuming, a computational model is developed for calculation of the two optimal angles. This combination is then verified in the irradiation geometry using EBT-3 GafChromic films (Ashland Advanced Materials, New Jersey) in an anthropomorphic disc phantom.

### 2.1 Workflow

For TSEI, the target volume is defined as the entire skin surface to a subcutaneous depth of 10–12 mm. In addition, the plaques are boosted with individual electron fields. For complete target volume coverage multiple irradiation fields of 6 MeV electrons are required. Extension of the plaques and their depth is determined in a CT scan. Energy of the electron boost fields is chosen according to the depth of the corresponding plaque. For treatment preparation, the number of fields with fixed monitor units per field are defined in the R&V system Mosaiq (Elekta, Stockholm). As part of the QA workflow, the fields are checked and approved by a second physicist.

Before the first irradiation of the patient a special quality assurance is carried out in which the consistency of the output and the profiles relative to the implementation is guaranteed (see section 2.4). In addition, the required accessories are checked for completeness: acrylic scatter plate, polystyrene blocks, eye protection, gloves with lead plates for shielding fingernails, toenail cover with lead, lead testicle protection, HDRE key and the HDRE frame. The initial setting as well as the subsequent irradiations take place in the presence of an expert senior physician, an experienced RTT, and a medical physics expert.

Irradiation is carried out with an electron beam with a nominal energy of 6 MeV in a HDRE mode. The $R_{50}$ is measured experimentally to $R_{50}=2.35\;\text{cm}$. With the formula of Harder $E_{0}=1.63+1.89R_{50}+0.019R_{50}^{2}$ [10] a mean energy at the surface of $E_{0}=6.17\;\text{MeV}$ can be calculated. A slide-in frame coded only for HDRE irradiation is used, allowing a field size of 40 × 40 cm^2^ at SSD = 100 cm from the focus or, after intercept theorem, a field size of 132 × 132 cm^2^ in patient position of SSD = 330 cm. The field size at patient's position is depending on gantry angle. The margin above feet or head is more than 40 cm.

In treatment position the patient stands 3.30 m away from the linac's focus on a polystyrene structure, about 16 cm high so that the umbilicus is approximately at the level of the central beam at gantry 270°. The elevated position of the patient is due to backscattering of electrons from the floor that could lead to an undesired dose increase on the lower legs [5]. To shift the maximum dose towards the surface of the skin, a 5 mm acrylic scatter plate is placed around 50 cm in front of the patient. Because of energy loss in the 5 mm thick acrylic scatter plate, the mean energy of the electrons is degraded. A $R_{50_{AS}}=1.65\;\text{cm}$ measured behind the acrylic wall corresponds to a mean energy $E_{0_{AS}}=4.17\;\text{MeV}$. The energy loss of 1.37 MeV is in the same order as written in Müller et al. [3].

For the shape and construction of this wall, various versions existed in the past [11]. So far, a wall with 4 segments with corresponding abutting edges was used as an alternative for the originally reported wall in the form of a half-cylinder [12].

**Figure 1:**
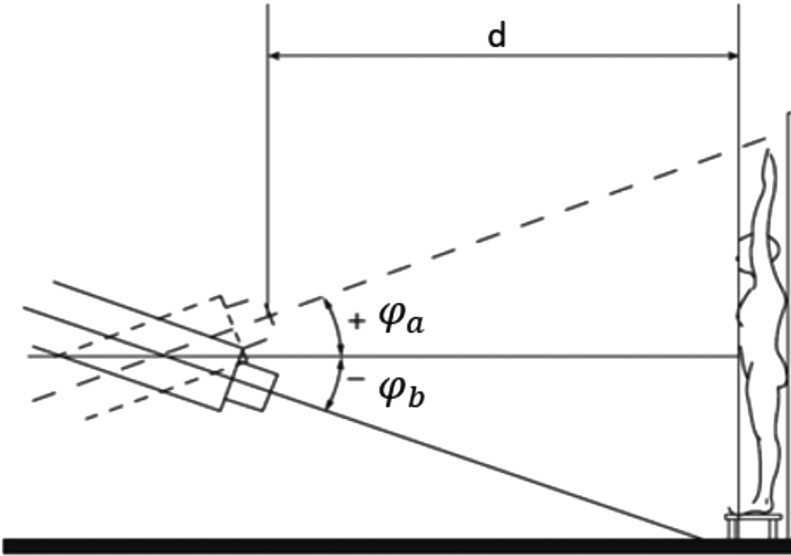
TSI positions [13], [14].

**Figure 2:**
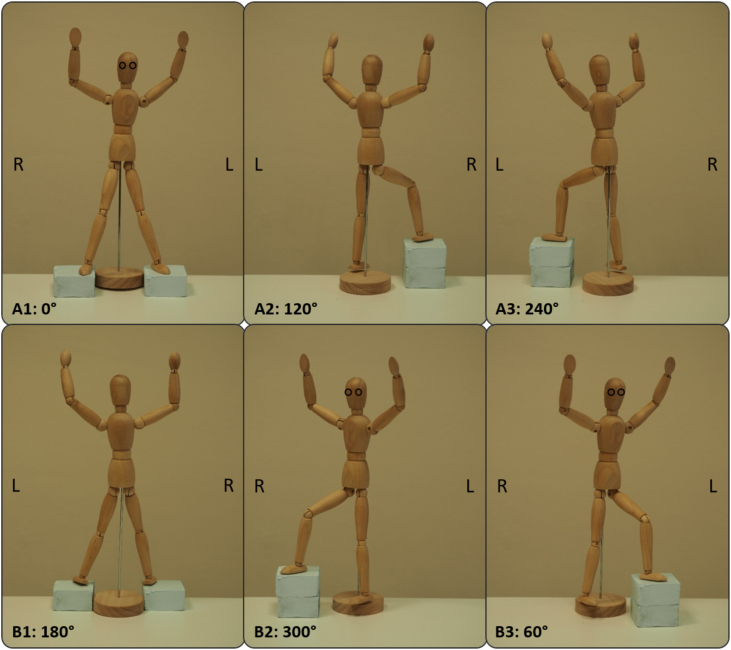
Upper positions (A1, A2, A3) are irradiated with both fields (a, b according to sketch above) on Mondays + Thursdays, lower positions (B1, B2, B3) on Tuesdays + Fridays. The degree label indicates the rotation of the patient around his own axis. At 0° the patient is facing the gantry. Any further + 60° position is indicated clockwise from the patient's point of view. L and R stand for left and right from the patient's point of view.

For a stable posture, the patient holds on to a device that can be adjusted to different body sizes using a telescopic extension with a snap-in function every 60°, defining the different positions. To achieve a homogeneous dose distribution in the entire skin surface the patient is positioned in six different postures relative to the radiation field [5]. Patient setup is illustrated in Figure 1. In analogy to the Stanford method, two axial fields with a single dose of 1.5 Gy are used in combination with these six patient positions. Patient positions A1–A3 are irradiated with both fields on Mondays and Thursdays; positions B1–B3 on Tuesdays and Fridays so that most parts of the skin receive 6 Gy after irradiation of all 6 positions (Figure 2). Skin regions that are not covered by this technique are irradiated once a week (on Wednesdays) with 6 MeV electron fields in regular dose rate mode (saturation fields). Due to the scattered radiation from the main fields, the incident fields already receive a part of the dose. Thus, the vertex and shoulder only need to be saturated with 18 Gy to reach a total dose of 30 Gy. In the submammary, sole of foot, anal fold, and palm regions, it can be assumed that almost no dose will arrive, which is why these regions are saturated with 24 Gy. Since the acrylic scatter plate is not used in the saturation fields, other bolus material with suited thickness is used to get more dose in the surface direction. Formable lead is used in all irradiations to protect sensitive body regions (eyes, toenails, and fingernails (starting at 18 Gy), testicles).

### 2.2 Calculation of treatment field angles

**Figure 3:**
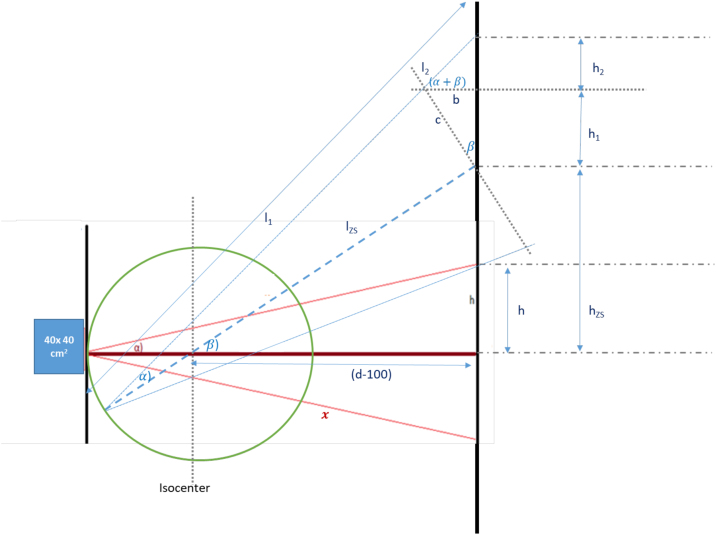
Theoretical basis for longitudinal profile calculation with any gantry angle.

To avoid numerous measurements, the optimal gantry angles $\varphi_{a}$ and $\varphi_{b}$ for a homogeneous dose profile along the vertical axis *h* were calculated using trigonometric functions based on measurement data in reference position at a gantry angle β = 270° (see Figure 3):

• In reference conditions (β = 270°, d = 330 cm) dose $D_{ref}\left(h,270\right)$ along the vertical axis h (−100 cm ≤ h ≤ 100 cm) was measured for 50 MU in steps of 10 cm using a Farmer chamber (30010, PTW, Germany), see Figure 3 red line.

Dose $D\left(h,\beta \right)$ was calculated based on these initial measurements: $D\left(h,\beta \right)=D_{ref}\left(h,270\right){\frac{l_{ref}}{l}}^{-k}$ with *k* *=* 2.18 describing the dose-distance dependence $D\left(d\right)$ along the central axis that was determined by corresponding measurements. See Table 4 for details.

• The optimal gantry angles yielding a homogeneous dose coverage $D\left(h\right)$ when superimposing two fields were determined manually, see Figure 3 blue line.

At the reference position at gantry angle β = 270°, the model predicts the dose $D\left(d\right)$ for any other angle. The optimal angular positions were verified by measurement.

### 2.3 Dosimetry

#### 2.3.1 Beam data characteristic

To investigate the beam characteristics of the 6 MeV electrons of the HDRE mode, PDDs and longitudinal profiles are recorded, monitor calibration is performed at the standard distance (SSD = 100 cm) and an absolute output factor (MU/Gy ratio) is determined at a reference location in treatment geometry. In the process, the mean range R_50_ was read off from the PDD at the standard distance according to IAEA 398 [8], where the ionization chamber is positioned at a defined reference depth of 1.43 cm for the output measurements.

The relative measurements (profiles + PDDs) were performed in the Blue Phantom^2^ (iba dosimetry, Schwarzenbruck) with two CC013 chambers (iba dosimetry, Schwarzenbruck) as a reference at a SSD = 100 cm under a gantry angle of 0°. Furthermore, the absolute measurements for the monitor calibration under a gantry angle of 270° were carried out with a Roos chamber in the water phantom (model 41023, PTW, Freiburg). For a comparison of the beam properties, all measurements were performed with 6 MeV in standard as well as in HDRE mode.

To obtain information about the beam characteristics in the irradiation geometry at a SSD = 330 cm under a gantry angle of 270°, a PDD was recorded by point measurements with the Roos chamber at different depths (step size 2 mm). These relative measurements and absolute dosimetry were performed in the water phantom (model 41023, PTW, Freiburg).

The profile in the longitudinal axis of the body was first determined with ionization chambers (Roos chamber 34001 and Farmer chamber 30013 (PTW, Freiburg), and a MatrixxEvolution (iba, Schwarzenbruck)) in front of a backscatter body. The longitudinal profile was recorded at SSD of 330 cm over a height of 200 cm at 10 cm steps. The data obtained with the Farmer chamber were used for dose profile calculation (section 2.2). In addition to the reference conditions (β = 270°) measurements were acquired in the optimal gantry angle combination determined according to the calculation described in section 2.2.

#### 2.3.2 Acrylic scatter plate

The influence of the Acrylic scatter plate on the dose distribution was investigated. For this purpose, the previously used segment-based wall was first used for measurements.

**Figure 4:**
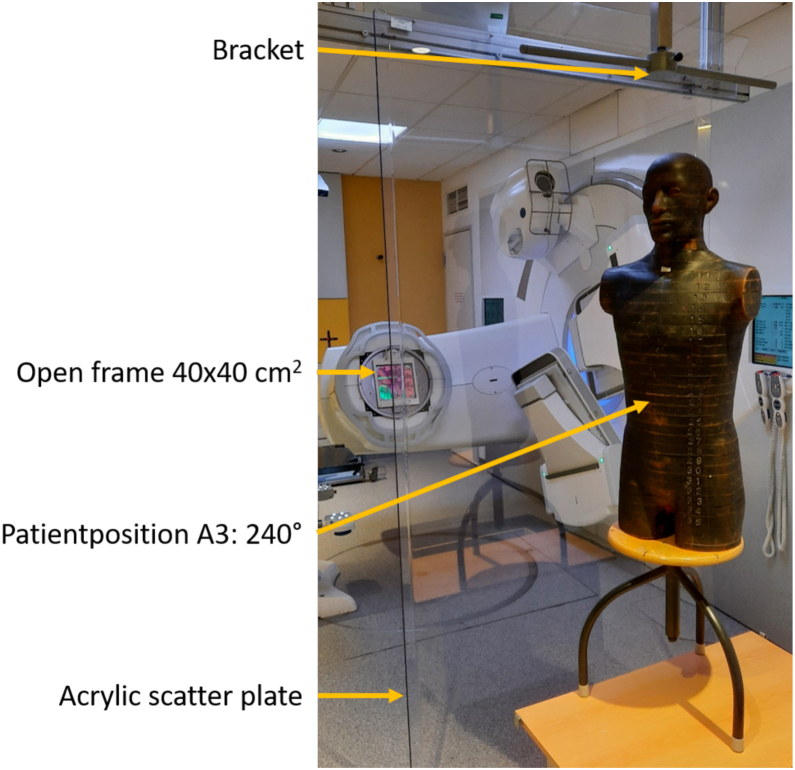
Positioning of anthropomorphic phantom with GafChromic EBT3 dosimetry films behind new acrylic scatter plate.

The old acrylic scatter plate consisted of four segments glued together, each 30 cm wide, 200 cm high, and 0.5 cm thick. Due to its semi-cylindrical shape, the acrylic scatter plate could stand without any other aids, but this meant that there was no fixed defined distance to the patient and the stability was rather poor. Furthermore, scattering effects occur at the production-related abutting edges of the segment-based acrylic scatter plate. This led to considerations to produce a new, planar acrylic scatter plate device (200 × 120 × 0.5 cm^3^), which is hung in a fixture suspended from the ceiling at a defined distance during treatment (see Figure 4).

Apart from the wall's shape the influence of the distance of the wall from the patient on the dose was investigated by placing both walls in front of the water phantom at different distances. The Roos chamber was then used to record a manual PDD and an absolute value for each distance.

#### 2.3.3 Film dosimetry

For additional validation of the treatment procedure and verification of the dose distribution in a measurement set-up as much as possible equivalent to the therapy setup, the homogeneity of the dose distribution was determined in an anthropomorphic disc phantom (meditron, Switzerland). As detector self-developing radiochromic GafChromic films (EBT-3, Ashland Advanced Materials, New Jersey) were inserted in-between the disks. The setup (see Figure 4) was then irradiated with the corresponding fields at the optimal gantry angles in the six different positions using 3 Gy per position.

To be able to assign a dose value to the film opacity in the disk phantom, a calibration curve was recorded at the reference setup (isocentre with electrons of 6 MeV at standard dose rate). For this purpose, monitor values from 30 MU to 600 MU were irradiated at 30 MU intervals onto a film from the same batch as the films in the phantom. At the same time, a Roos chamber was connected at the same depth to be able to assign a dose to the film opacity. The measurement depth was in the reference depth previously determined dosimetrically.

For exact dose evaluation, the films were digitized using an Epson Perfection C850 Pro scanner, whereby a 48-bit image with a resolution of 75 dpi was recorded. The evaluation was done with the GafChromic FilmQA Pro software (Ashland Advanced Materials, New Jersey).

### 2.4 Quality assurance for TSEI

As part of the quarterly absolute dosimetry, the 6 MeV are also measured in HDRE mode at a SSD = 100 cm with a gantry angle of 270°. For this, 30 MU are irradiated onto the Roos chamber, which is positioned at a depth of 1.43 cm (z_ref_ = (R_50_ − 0.6) [8]) in the water phantom 41023 (PTW, Freiburg). At this depth, 3 Gy should be achieved at 30 MU.

In addition to the absolute dosimetry, the homogeneity is checked on the day before the first TSEI irradiation of a new patient (expected about 2 patients per year). For this purpose, an array detector (MatrixxEvolution, iba, Schwarzenbruck) is positioned isocentrically on the irradiation table at SSD = 100 cm. At gantry = 0° the apertures are collimated without frame in service mode into a 20 × 20 cm^2^ field. Thus, a dose profile of 6 MeV can be recorded in HDRE mode. Furthermore, the value for 10 MU is noted by the physicists with a LinacCheck (PTW, Freiburg) and recorded as a reference.

Every day, on the TSEI irradiation days and starting shortly before, in addition to the standard electrons, the value for the 6MeV is recorded in HDRE mode with a LinaCheck by the medical-technical assistants.

### 2.5 Risk management

Since a new technique is being introduced at a new linear accelerator according to German legislation [15], a risk analysis must be carried out. For this purpose, the entire workflow that the patient undergoes with this irradiation technique is considered. The individual processes are described and possible failure modes assigned to each process point.

To be able to carry out a FMEA risk assessment systematically, in an initial step a process map (PM) was created for the TSEI workflow (see Table 1). This is to be read chronologically from top to bottom. The marked area describing physics treatment planning and treatment delivery is analysed in detail in the following.

**Table 1:** Process map (PM).

Workflow	Subtasks of the process			
Planning initial visit	Pre-registration initial meeting in the outpatient clinic	Review of documents	Organising diagnostic findings and summoning patients	Pre-therapy planning
Initial visit	*Information*: Therapy side effects	Physician's letter	*Medication*: Retinoids, Photosensitization	
Morning meeting	Introduction of the patient to all physicians			
Coordination	Allocation of dates	inpatient admission		
Imaging for treatment planning	CT for Boost	Photos of plaques		

Physical treatment planning	CT: to measure the plaques (Boost)	Standard beams in Mosaiq	Approval of beams	
Verification of treatment planning	Profiles PDDs (physicist)	Daily check of the HDRE dosimetry (therapist)		
Patient positioning of first treatment	With physics, RTTs and physician together	Explanation of the positions	*Protection material*: eye protection, testicle protection, finger protection	*Material*: acrylic scatter plate, Bracket, styrofoam
Treatment Total Skin	Patient is standing 3.30m from the isocentre	Styrofoam blocks, isocentre at navel height	*Monday/Thursday*: positions A1, A2, A3 *Tuesday/Friday*: positions B1, B2, B3	*Wednesday*: Saturation fields

Treatment Boost	Individual Plaque treatment			
Final visit	Final letter			
Follow up	After 6 weeks, then every 6 months			

A FMEA risk analysis is traditionally divided into a probability of occurrence (O), probability of detection (D) and severity (S) [16], [17]. Each point of a process is then evaluated according to a scale. In the evaluation a 5-point scale was used each.

Based on the process map, potential failure modes were determined by two medical physicists. The process map and the identified failure modes were then distributed to two additional physicists, three RTTs, and a senior physician with the task to i) identify potential additional failure modes and ii) to rate all failure modes according to O, D, and S. The risk priority number (RPN) was determined based on the median rating of each category.

According to the recommendations of the BfS, DEGRO, DGMP and DGN [18], the goal for all processes should be to achieve an RPN value of 4 (green). Furthermore, risks should only be accepted up to an RPN value of 15 (yellow). The maximum possible RPN value is 125 (red). Determined failure modes were grouped accordingly.

## 3 Results

### 3.1 Calculation of treatment field angles

**Figure 5:**
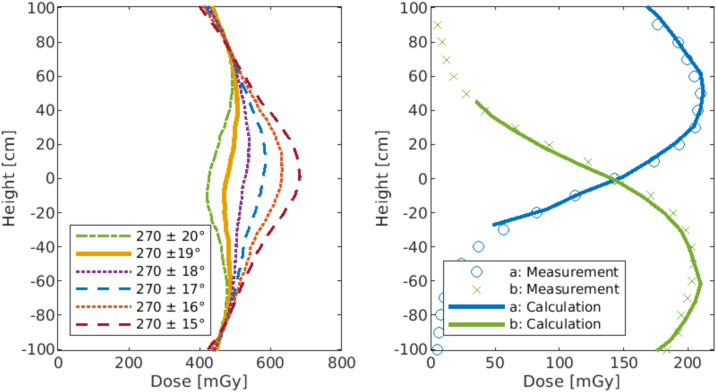
*Left image*: Influence of variations in gantry angle β on the dose homogeneity. Shown is the superposition of two fields with symmetric gantry angles about 270°. *Right image*: Comparison of measurement and calculation for the optimal gantry angle combination $\beta_{a}=289{}^{\circ}\;$ and $\beta_{b}=251{}^{\circ}$.

Figure 5 (left) shows the different calculated profiles in the range 270° ± [15°,20°]. The calculations yield that the combination of angles 270° ± 19° (289° and 251°) gives the most homogeneous profile. A combination of asymmetric angles (e.g. 270° + 19° and −18°) resulting in correspondingly asymmetric profiles was therefore excluded.

To confirm the calculated angles, the dose distribution for those longitudinal profiles was measured under the optimal angles. Measurement and calculation agree very well (Figure 5, right).

### 3.2 Dosimetry

#### 3.2.1 Beam data characteristic

**Figure 6:**
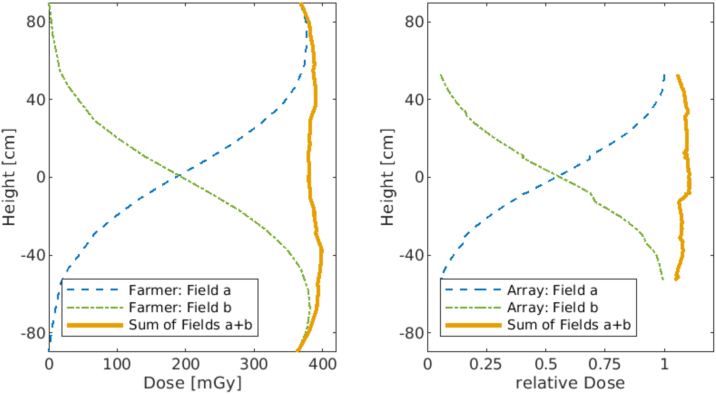
*Left image*: Relative axial dose distribution for two superposed standing fields. The distribution of the individual fields is shown separately. Measurement was performed with a farmer chamber. Data points were interpolated to guide the eye. *Right image*: Measurement was performed with a Matrixx.

The superposition of two standing fields with ±19° vertical deflection, results in a balanced dose distribution at a focus-skin distance of 330 cm (Figure 6, left). The orange curve shows the sum of the two dose profiles, which has a homogeneity of ±10% along the patient's longitudinal axis. Figure 6 (right) shows the data of the same setup but was measured with an array. The peaks in the profile of the Matrixx can be explained by errors in the positioning of the matrix. Since no absolute dose values were recorded with the array, the relative measured dose is shown.

**Figure 7:**
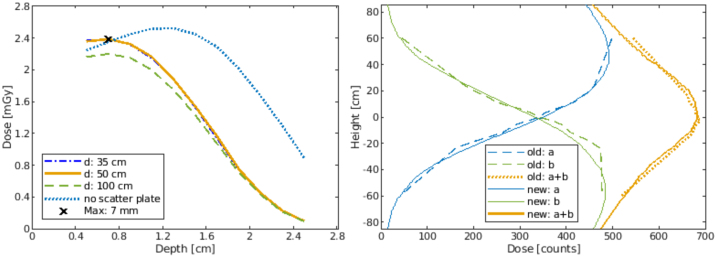
*Left image*: PPDs of the same energy spectrum, but with variable distance of the Acrylic scatter plate to the measuring chamber. Due to the energy loss in the 5 mm thick acrylic scatter plate the mean energy at the surface $E_{0}=6.17\;\text{MeV}$ plate (blue dotted line) is degraded to $E_{0_{AS}}=4.8\;\text{MeV}$ (orange solid line). *Right image*: ±15° longitudinal profiles and their sums. Dashed line profiles with old segmented acrylic scatter plate, solid line profiles with new plane acrylic scatter line.

The reference conditions in treatment geometry were set as follows: SSD = 330 cm, gantry angle 270°, Roos chamber in 7 mm water depth (Figure 7, left) – equivalent to 12 mm effective depth minus 5 mm thickness of acrylic scatter plate. In the final set-up, the maximum dose at 7 mm can be seen in the PDD. The required monitor values (absolute output factor) for the single therapy dose of 1.5 Gy were determined, to 200 MU.

#### 3.2.2 Acrylic scatter plate

Figure 7 left shows the PDD without acrylic scatter plate (blue) and the PDDs with the plate at different distances. In the range 35–50 cm distance of the acrylic scatter plate to the patient there is almost no difference. At a distance of 100 cm, the dose is about 0.3 mGy lower at the maximum due to the scattering effects.

The influence of the production-related abutting edges in the Acrylic scatter plate is visible dosimetrically in the transverse profile, which makes reproducible positioning difficult (Figure 7 right, green dashed line).

To ensure reproducible positioning and homogeneous irradiation, the construction of the acrylic scatter plate was changed. A flat plate was made, which prevents unwanted scattering effects at the edges. The new acrylic scatter plate is fixed to the ceiling at a distance of 50 cm in front of the handle ensuring proper patient positioning (Figure 4).

#### 3.2.3 Film dosimetry

**Figure 8:**
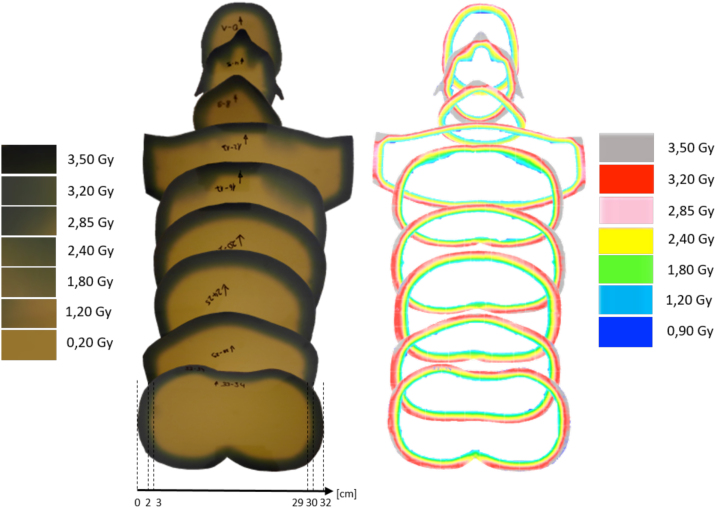
Scanned films from the anthropomorphic disc phantom. *Left image*: homogeneous blackened ring of Gafchromic films. *Right image*: Total skin dose distribution in the Rando Phantom, evaluated with the FilmQA Pro software.

The films from the anthropomorphic disc phantom were blackened at the circumference of the phantom after irradiation in patient geometry (Figure 8, right). The blackening decreases from the outer edge and disappears after 2–3 cm inwards (depending on the body region). This corresponds to the range of the 6 MeV electrons at 80% of the PDD measured at a distance of 100 cm in the large water phantom (therapeutic range = 2 cm) and with a practical range of the 6 MeV electrons of about 3 cm.

After digitization of the films, the calibration, curve was used to convert opacity to a dose distribution. Compilation and dosimetric analysis of all evaluated films with their isodoses is shown in Figure 8 on the left.

Most layers have a homogeneous edge of 3 Gy ± 15%. It is noticeable, however, that in the second layer the regions for the tip of the nose, paranasal sinuses, and the ears are overdosed by up to 15%.

#### 3.2.4 Quality assurance for TSEI

Constancy test characteristics were defined. A corresponding task for the total skin technique was created in myQA (iba, Schwarzenbruck) to regularly check the constancy tests required for the total skin technique (daily, quarterly, annually before the first RT).

### 3.3 Risk management

The result of the FMEA analysis is shown in Table 2. The highest RPNs were determined for the failure modes “wrong fields released” with a value of 20 and “position irradiated twice” with a value of 30. Applying the recommendations of BfS [18] results into 1/11/3 failure modes in categories green/yellow/red, respectively.

**Table 2:** Failure modes and the median values of the expert opinion for severity (S), occurrence (O) and detectability (D) for each process step and the corresponding subtask according to Table 1 RPN = risk priority number; TSI = total skin irradiation, Sat.: saturation fields.

Process step	Subtask	Failure mode	S	O	D	RPN
Physical treatment planning	CT: to measure the plaques (Boost)	Determine incorrect thickness of the plaques → *Wrong energy* (Saturation)	2	2	2	8
	Standard beams in Mosaiq	*Wrong fields*/energy/MU/gantry angles	TSI: 5 Sat.: 3	TSI: 1 Sat.: 2	TSI: 2 Sat.: 2	TSI: 10 Sat.: 12
	Enable beams	*Wrong fields*, Incorrect designation, double/not at all, unconcentrated control	TSI: 5 Sat.: 3	TSI: 2 Sat.: 2	TSI: 2 Sat.: 2	TSI: 20 Sat.: 12
Verification of treatment planning	Profiles, PDDs (physicist)	Wrong SSD, wrong energy	3	2	1	6
	Daily check of the HDRE dosimetry (therapist)	Wrong output	3	1	2	6
Patient positioning of first treatment	With physics and doctors together	Disagreement	2	1	1	2
	Explanation of the positions	Misunderstanding between patient and employee	3	1	2	6
	*Protection material*: eyes, testicle, fingers	Forgetting or slipping of the protective clothing	3	2	2	12
	*Material*: acrylic scatter plate, Bracket, styrofoam	Forgetting material or broken bracket	3	2	2	12
Treatment Total Skin	Patient is standing 3.30 m from the isocentre	Other distance/misplacement	4	1	2	8
	Styrofoam blocks, isocentre at navel height	Wrong height	2	3	3	18
	*Monday/Thursday*: positions A1, A2, A3 *Tuesday/Friday*: positions B1, B2, B3	position forgotten/doubled/wrongly delivered fraction	5	2	3	30
	*Wednesday*: Saturation fields	Wrong energy/wrongly delivered fraction	3	1	3	9

Looking at the highest RPN of each process step in more detail reveals that the most crucial step in treatment planning is connected to a wrong choice and more important to a missing detection of a wrong choice of treatment parameters in plan approval. TSEI plans consist of identical treatment fields according to the optimized settings described above. An undetected wrong setting of energy, monitor units or gantry angles could lead to severe (S = 5) side effects especially for the total skin but also for the saturation fields.

At the first treatment fraction which always takes place with a senior physician, a MPE and an RTT, the highest RPN was attributed to protection material. Missing protection aids for eyes, testicles or fingernails in this first fraction might lead to missing protection in all remaining fractions and bears thus the risk of, e.g. falling-off fingernails.

For the treatment as a process step itself the highest risks are related to mixing up treatment fields and patient positions, e.g., doubling of certain combinations (RPN = 30).

Table 3 shows the differences in risk assessment between the TSEI solution implemented until 2019 at the Primus linac (Siemens) and the current implementation at the VersaHD (Elekta). Only the first two items are critical, which include a different monitor value and the fixed gantry angle positions. The other differences lead to a much safer irradiation.

**Table 3:** Changes in failure modes comparing previous TSEI solution at a Primus linac (Siemens) to the current standard using a VersaHD (Elekta).

Primus	Versa HD	Possible error	S	O	D	RPN
1300 MU = 1.5 Gy	200 MU = 1.5 Gy	Old MU value in RT field → 6.5× higher dose	3 → 5	2 → 2	2 → 3	12 → 30
Individual Gantry angles	Fix Gantry angles	Don‘t use optimal calculated beams	4 → 3	2 → 3	4 → 3	32 → 27
Distance Gantry – Patient: 2.85 m	Distance Gantry – Patient: 3.30 m (fixed holder)	No acrylic scatter plate	2 → 1	3 → 1	4 → 1	24 → 1
Open frame 30 × 30 cm^2^; all frames possible Normal key for all treatments Normal machine for all treatments	Only Open frame 40 × 40 cm^2^ Only HDRE key Only 6 HDRE with VersaHD machine	Less wrong applicator possible Less errors possible No wrong energy/machine possible	2 → 1	1 → 1	1 → 1	2 → 1

**Table 4:** Theoretical consideration of the gantry angle. With: d = 330 cm measuring distance, β: = gantry angle, α: = divergence angle, *M*_*n*_: Measurement value per measurement position n of the longitudinal profile over a length of 200 cm at gantry 270°, *k*: Exponent describing the distance dependence of the dose; is determined experimentally.

Designation	Formula
Height of the angled beam h_ZS_	$h_{ZS}=\left(d-100\right)\;tan\;\beta$
Beam length l_ZS_	$l_{ZS}=100+\frac{d-100}{\cos\;\beta }$
Height c of the angled beam	$c=l_{ZS}\;\tan\;\alpha$
Determination of b	b=c sin β
Beam length l_1_	$l_{1}=\frac{l_{ZS}}{\cos\;\alpha }$
Beam length l_2_	$l_{2}=\frac{b}{\cos(\alpha +\beta )}$
Beam length l	$l=l_{1}+l_{2}=\frac{l_{ZS}}{\cos\;\alpha }+\frac{l_{ZS}\;\sin\;\alpha \;\sin\;\beta }{\cos\;\alpha \;\cos(\alpha +\beta )}$
Reference beam length *x*	$x=\frac{d}{\cos\;\alpha }$
Projection onto the longitudinal axis h_1_	$h_{1}=c\;\cos\;\beta$
Projection onto the longitudinal axis: h_2_	$h_{2}=b\;\tan(\alpha +\beta )$
Projection onto the wall h	$h=230\;\tan\;\beta \pm l_{ZS}\;\tan\;\alpha \;\cos\;\beta \pm l_{ZS}\;\tan\;\alpha \;\sin\;\beta \;\tan\left(\alpha \pm \beta \right)$
Distance corrected dose D per measuring position n	$D=\frac{M_{n}}{{\left(\frac{l}{x}\right)}^{k}}$

## 4 Discussion

The conversion of the total skin technique to a new accelerator could be carried out with only few changes and a risk analysis as requested by the authorities. The determined gantry angles lead to a homogenous coverage of the skin. The optimized geometry of the acrylic scatter plate further improves the homogeneity. Among the changes is an optimized setup w.r.t. backscatter from the floor as proposed by Nevelski et al. [5]. Multiple polystyrene blocks on the floor are now foreseen to bring the patient's navel to isocentre height. Based on the PDD in SSD = 100 cm, a bremsstrahlung background of approx. 2% can be estimated. Accordingly, for a single dose of 3 Gy, this would mean a total of approx. 0.18 Gy bremsstrahlung background for all 6 positions. However, this measurement was not repeated at full depth at an SSD = 330 cm.

One weakness of the measurements is that the rack for measuring the longitudinal profile could not be positioned with millimeter accuracy. Besides, due to the predefined levels, it was not possible to carry out a smooth transition for the measurements with the MatrixxEvolution. An effect of the slightly inaccurate positioning of the MatrixxEvolution can be seen in Figure 6 on the right. Because the rack has a forward slope, a seamless transition is no longer possible after a certain position. This is also a reason why a Farmer Chamber was used for the final measurements instead of the MatrixxEvolution.

Compared with the papers of Schiapparelli et al. [1] and Nevelesky et al. [19], the results can be confirmed in the proposed treatment methodology. We also concluded that the horizontal gantry angles should have an offset of ±19° to assure a homogenous coverage at the distance of 3.30 m required for our setup. Slightly different is further the position of the permanently installed bar for ensuring the six patient positions (280 cm in our setup, 353 cm in Schiapparelli et al. [1]) and the thickness of the acrylic plate (5 mm in our setup, 10 mm in Schiapparelli et al. [1]). Since both validation measurements show feasibility for TSEI, those parameters can be chosen with some flexibility which is advantageous since often local constraints such as other mechanical installations have to be considered.

With the simple multi-field technique with two axial fields and six patient positions, a sufficiently homogeneous dose is applied to the skin, which is hardly inferior to continuous patient rotation [3]. Underdosage is only observed in the strongly concave body contours and are saturated with additional standing fields once a week. Overdosage of ∼15% were observed close to the nose and ears in the film measurements. Since the ears protrude further from the body than other organs, they are covered by more than just two patient positions, which may explain these overdoses. The air in the sinuses causes the electrons to penetrate deeper, which explains the overdoses there. The other narrow overdoses at the edge of the body can be explained by not quite plane transitions between the layers of the phantom.

Risk analysis followed the in-house rules of the University Clinic as well as the TG 100 and the FMEA TSEI paper by Ibanez-Rosello et al. [17]. In TG 100, a 10-level scale is proposed, just as in Su et al. [20], [21], whereas in Ibanez-Rosello et al. and also by internal regulations a 5-level scale is used. Risks of the methodology of the old linear accelerator were estimated retrospectively since formal risk analyses are carried out in our clinic only recently. Compared to those risks, the new methodology could reduce risks by optimizing a field setup and patient positioning allowing that every patient can be treated with identical gantry angles and the same monitor values. In addition, the permanent mount of Acrylic scatter plate and handle for patient positioning reduces risk for mispositioning compared to the mobile solutions in the previously used methodology (from 24 to 1, see Table 3). However, during the transition phase of introducing the new method to the clinic, there is a very high risk that the monitor values of the old accelerator (1300 MU) will be entered for the new accelerator (200 MU). These are now lower by a factor of 6.5, which means that in the worst case, the patient could receive 195 Gy instead of 30 Gy over the entire treatment with a high risk of severe side effects. As a measure for this, a structured instruction was created, which is to be worked through step by step by RTTs and physicists and must be checked additionally by another person in each case. Compared to other publications, the new monitor value is in the middle range. In Schüttrumpf et al. [22] the value ranged between 117 MU and 282 MU.

For the introduction of the new total skin technique, the specified RPN is given as a starting value. After a few therapies, the values will be checked and adjusted. The RPNs rated “red” only mean that special attention must be paid to these steps. According to this, the following points would be particularly susceptible to errors: the incorrect creation of the standard fields in the R&V Mosaiq and their corresponding incorrect approval when the patient is standing directly on the floor and when a patient position is irradiated twice or not at all during irradiation. To be able to validate whether the procedure is correct, it is planned to introduce check lists. After an appropriate period the risk analysis will be repeated to reassess all failure modes.

## 5 Conclusions

The modified Stanford technique with 6 patient positions was adapted to the new linear accelerator. According to AAPM guidelines, TG-142 and IAEA TRS-398, relative and absolute measurements were carried out, giving a consistent result. Using a simple calculation model, the two optimal angles in superposition for a homogeneous profile could be determined. Since the treatment is rarely carried out, a simple and easily reproducible technique was necessary. This is possible with a plane acrylic scatter plate in a defined distance and a holder which locks in 6 equidistant positions. The patient stands at a distance of 3.30 m from the linac isocenter during irradiation. Furthermore, quality assurance was implemented, which has to be performed by the physicists on the day before the first irradiation and by the RTTs daily. The FMEA-based risk analysis showed, that due to various circumstances the TSEI bears risks for severe damages. They can be controlled by use of appropriate methods and in comparison to the methodology at the previously used linac, the overall risk could be reduced by the presented new implementation.

## Declaration of interests

The authors declare that they have no known competing financial interests or personal relationships that could have appeared to influence the work reported in this paper.

## Conflicts of interest

The authors have no relevant conflicts of interest to disclose.
